# Loss of IL10RA rewires epithelial immune responses to intracellular *Staphylococcus aureus* small colony variants in bovine mammary epithelial cells

**DOI:** 10.3389/fcimb.2026.1891987

**Published:** 2026-09-14

**Authors:** Umesh K. Shandilya, Yan Liang, Heba Atalla, Bonnie Mallard, Niel A. Karrow

**Affiliations:** 1 Department of Animal Biosciences, University of Guelph, Guelph, ON, Canada; 2 College of Animal Science and Technology, Yangzhou University, Yangzhou, China; 3 Department of Pathobiology, Ontario Veterinary College, University of Guelph, Guelph, ON, Canada

**Keywords:** bovine mastitis, CRISPR/Cas9, IL10RA, MAC-T cells, RNA-Seq, small colony variant (SCV), *Staphylococcus aureus*

## Abstract

**Introduction:**

*Staphylococcus aureus* small colony variants (SCVs) represent a persistence-adapted phenotype linked to chronic and recurrent bovine mastitis. Interleukin-10 receptor ⍺ (IL10RA) is a central regulator of anti-inflammatory signalling in the mammary gland, but its role in shaping transcriptional responses to intracellular staphylococcal challenge has not been characterized.

**Methods:**

CRISPR/Cas9-generated IL10RA knockout (KO) MAC-T bovine mammary epithelial cells (MECs), alongside wild-type (WT) controls, were infected with either *S. aureus* SCV strain Heba3231 or its isogenic parental strain (PS). Transcriptional responses were profiled by RNA sequencing and compared across genotype and infection conditions.

**Results:**

WT cells mounted markedly divergent responses to PS (8 DEGs) versus SCV (461 DEGs) infection, with no overlap between the two gene sets, indicating strain-specific engagement of distinct molecular programmes. SCV infection of WT cells up-regulated lipid and sterol biosynthetic pathways while suppressing epithelial barrier integrity genes (*S100A8*, *S100A9*, *TGM3*, *KRTDAP*). IL10RA disruption substantially amplified transcriptional dysregulation: *IL10RA*-KO cells infected with PS yielded 619 DEGs (a 77.4-fold increase over WT-PS), while *IL10RA*-KO cells infected with SCV yielded 1,297 DEGs (a 2.8-fold increase over WT-SCV). Approximately 32 to 36% of IL10RA-regulated genes formed a shared core across infection conditions. Loss of IL10RA derepressed pro-inflammatory cytokine pathways (TNF, IL-17), enhanced pro-apoptotic programmes during SCV infection, and suppressed phagosomal maturation and lipid metabolic gene programmes. Candidate IL10RA-responsive genes, including *CD79B, WFDC2, AMN* and *LY6E*, were identified as targets for further validation.

**Discussion:**

These findings establish IL10RA as a broad transcriptional homeostasis regulator in bovine MECs during staphylococcal infection, with disproportionate effects on inflammatory and barrier-related gene programmes under SCV challenge. The identified candidate genes warrant validation in primary bovine mammary cells, milk somatic cells, or clinical mastitis samples to confirm their relevance to chronic and recurrent disease pathogenesis.

## Introduction

Bovine mastitis remains one of the most economically devastating diseases affecting the global dairy industry, imposing annual losses estimated in the billions of dollars through reduced milk production, increased treatment costs, premature culling, and compromised animal welfare. *Staphylococcus aureus* is an important agent of contagious bovine intramammary infections in dairy cattle, with its persistence inside the udder underpinned by mechanisms including biofilm formation, polysaccharide capsule production, and the capacity to invade both professional and non-professional phagocytes (Zaatout et al., 2020). These properties shield the pathogen from the host’s innate and adaptive immune response as well as from antibiotic therapy, rendering *S. aureus* mastitis notoriously refractory to treatment (Ban-Cucerzan et al., 2026).

A particularly insidious adaptation associated with chronic and recurrent mastitis is the formation of *S. aureus* small colony variants (SCVs). Persistent or difficult-to-treat *S. aureus* infections in animals and humans may be associated with SCVs that can hide within host cells and modulate host defenses (Atalla et al., 2011). SCVs form small, colorless, non-hemolytic colonies, exhibit a slow growth rate and prolonged lag phase, and show reduced expression of virulence factors such as agr, hla, and coagulase, alongside increased expression of fermentation pathway genes (Atalla et al., 2008). The bovine SCV Heba3231, isolated from a cow with a history of chronic mastitis, represents a well-characterized naturally occurring model strain (Atalla et al., 2008). Persistent bovine *S. aureus* mastitis is attributable to the versatility of this pathogen within the mammary gland environment, and the formation of SCVs that can survive within host cells; viable SCV Heba3231 were detected within a bovine mammary epithelial cell (MEC) line (MAC-T) for up to 96 hours post-infection, while its parental strain 3231 was not recoverable at 24 hours or later (Atalla et al., 2010a). Marked differences exist in the immune responses induced by parent and SCV forms of the same *S. aureus* strain, highlighting the distinct immunological challenges posed by the SCV phenotype in the context of persistent bovine mastitis (Atalla et al., 2010b).

Bovine MECs serve as critical sentinel cells at the host–pathogen interface. Pathogen-recognition by MECs elicits the release of inflammatory mediators that help activate the mammary gland’s innate defense mechanisms. Following recognition of microbial-associated molecular patterns (MAMPs) by pattern recognition receptors (PRRs) such as toll-like receptors, downstream signal transduction leads to the activation of transcription factors that regulate cytokine, chemokine, and antimicrobial peptide gene expression (Faaz and Abdullah, 2022). The transcriptional response of these cells to *S. aureus* differs substantially from that elicited by Gram-negative pathogens, with *S. aureus*-derived MAMPs activating the transcription factor AP-1 and the interleukin (IL)-17A signaling pathway in MECs, in contrast to the transcription factor NF-κB, and Fas signaling pathway activation characteristically associated with bacterial lipopolysaccharides (Gilbert et al., 2013). Understanding how intracellular *S. aureus* SCVs specifically modulate mammary epithelial transcriptional programs remains an area of active investigation.

Central to the regulation of the mammary gland inflammatory response is the anti-inflammatory cytokine IL-10. When this cytokine homodimer binds to the IL-10 receptor α (IL-10RA) extracellular domain, it initiates oligomerization with IL-10 receptor β (IL-10RB), and the subsequent phosphorylation of Janus Kinase 1 (JAK1), Tyrosine Kinase 2 (Tyk2), and signal-transducer and activator of transcription 3 (STAT3). After dimerization, STAT3 translocate to the nucleus, initiating the transcription of target genes, including suppressor of cytokine signaling 3 (SOCS3), that inhibits NF-κB nuclear translocation and the expression of pro-inflammatory genes (Carlini et al., 2023). Cows with mastitis exhibit significantly higher levels of *IL-10* gene expression compared to healthy animals, consistent with IL-10’s role in inhibiting NK cells, macrophages, and Th1 cell production of IL-1 and tumor necrosis factor (TNF) α (Faaz and Abdullah, 2022).

The gene encoding IL-10RA (IL10RA) has emerged as a significant candidate gene in the context of bovine mammary health. Genetic variation in the form of single-nucleotide polymorphisms (SNPs) in genes regulating anti-inflammatory responses, including *IL-10Rα*, can influence an animal’s susceptibility to acute or chronic mastitis. Six SNPs were identified in bovine *IL-10Rα* and found to be associated with estimated breeding values for somatic cell score, a trait highly correlated with mastitis incidence (Verschoor et al., 2009). Functional studies using CRISPR/Cas9-generated IL10RA-knockout (KO) (MAC-T cells have further illuminated this gene’s biological significance. In comparison to wild-type (WT) cells stimulated with *Mycobacterium avium* subsp. *paratuberculosis* (MAP) cell lysate, IL10RA-KO MAC-T cells exhibited upregulation of pro-inflammatory cytokine gene expression, including *TNFA*, *IL1A*, *IL1B* and *IL6*, downregulation of *SOCS3*, and elevated secretion of TNF-α, IL-6, IL-8, CCL2, and CCL4 (Mallikarjunappa et al., 2020). Subsequent MAP infection of IL10RA-KO MAC-T cells demonstrated greater secretion of TNF-α, IL-6, IFN-γ, CCL3, CCL4, CXCL8 and CXCL10, and further revealed that IL10RA appears to be involved in regulating miRNA-mediated immune responses to this intracellular pathogen (Shandilya et al., 2023).

RNA sequencing (RNA-seq) provides powerful tools to investigate global gene expression changes associated with host-pathogen interactions. By combining CRISPR-mediated gene editing with RNA-seq, it is possible to systematically evaluate the role of specific genes, such as IL10RA, in regulating host responses to infection at a genome-wide level.

Despite these advances, the role of IL-10RA in regulating the transcriptional response of bovine MECs to intracellular S. aureus SCV challenge remains unknown. Because the SCV phenotype can persist intracellularly for extended periods and differs immunologically from its parental strain, defining IL10RA-dependent transcriptional responses may provide insight into host pathways associated with SCV infection and persistence, while functional effects on bacterial survival remain to be determined. In this study, we aimed to investigate the role of IL10RA in regulating transcriptional responses of bovine MECs to *S. aureus* SCV infection under conditions previously shown to support bacterial internalization. Using CRISPR-generated IL10RA-KO MAC-T cells and their WT counterparts, we performed RNA-seq analysis following infection with *S. aureus* SCV and its isogenic parental strain. We hypothesized that IL10RA deficiency would significantly alter host immune and inflammatory pathways, thereby influencing the cellular response to SCV infection. Understanding these mechanisms may generate candidate genes and pathways for future validation in more physiologically relevant bovine mammary models and clinical samples.

## Materials and methods

### Maintenance of IL10RA-KO and wild-type MAC-T cells

An IL10RA-KO bovine MEC line was previously generated in our laboratory using CRISPR/Cas9-mediated genome editing in MAC-T cells. The generation and validation of this IL10RA-KO MAC-T cell line have been described previously by Mallikarjunappa et al. (2020). Briefly, IL10RA disruption was confirmed in the original KO line using Sanger sequencing and a Western blot and the functional characterization demonstrated altered IL-10/IL10RA-associated immune responsiveness relative to WT MAC-T cells, including increased pro-inflammatory cytokine gene expression, reduced SOCS3 expression, and elevated secretion of inflammatory mediators following immune stimulation. The same validated IL10RA-KO MAC-T cell line was used in the present study. The corresponding WT MAC-T cells were maintained in parallel as controls.

Both WT and IL10RA-KO MAC-T cells were cultured in Dulbecco’s Modified Eagle Medium (DMEM) supplemented with 10% fetal bovine serum (FBS) and 1% penicillin–streptomycin (100 U/mL; Invitrogen, Thermo Fisher Scientific). Cells were incubated at 37 °C in a humidified incubator with 5% CO_2_ and sub-cultured routinely upon reaching approximately 80-90% confluency to ensure optimal growth conditions.

### Bacterial strains and preparation

Two well-characterized *S. aureus* strains were used for infection experiments: the small colony variant (SCV) Heba3231, and its isogenic parental strain (PS) 3231, both originally isolated and characterized by Atalla et al. (2008, 2010a) and maintained in the Department of Pathobiology, Ontario Veterinary College, University of Guelph. For each independent experiment, bacterial stocks preserved at -80 °C were revived and cultured freshly. Bacteria were grown in an appropriate medium to the early exponential phase before infection. Optical density at 600 nm (OD_600_) was adjusted to 0.2 for SCV cultures and 0.4 for parental strains to account for growth differences between phenotypes. Bacterial cells were harvested by centrifugation (1000 × g for 20 min at 4 °C), washed three times with sterile phosphate-buffered saline (PBS, pH 7.4), and resuspended in complete DMEM to achieve the desired bacterial concentration for infection.

### *In vitro* infection assay

The WT and IL10RA-KO MAC-T cells were seeded into 24-well tissue culture plates at a density of 5 × 10^4^ cells per well and allowed to adhere and proliferate until reaching approximately 80% confluence. Cells were subsequently challenged with either the SCV, or parental *S. aureus* strains at a multiplicity of infection (MOI) of 25:1 (Atalla et al., 2010a). Uninfected cells, receiving an equivalent volume of sterile culture medium, served as negative controls. To enhance bacterial contact with epithelial cells, plates were briefly centrifuged at 250 × g for 2 min immediately following inoculation. Infection was carried out for 3.5 ah at 37 °C in a 5% CO_2_ incubator. The 3.5 ah infection period was selected to align with the established *in vitro* MAC-T infection model for this isogenic SCV/PS strain pair at MOI 25:1 (Atalla et al., 2010a), and to capture the initial epithelial transcriptional response to bacterial internalization before the infection trajectories of the two phenotypes diverge substantially. Specifically, PS 3231 is not recoverable by 24 ah, whereas SCV Heba3231 can persist intracellularly for up to 96 ah (Atalla et al., 2010a); therefore, sampling both strains at a common early time point enabled a direct comparison of the initiating host response under matched infection conditions.

The infection conditions used here were based on the previously validated MAC-T infection model of Atalla et al. (2010a), in which bacterial internalization and persistence of SCV Heba3231 were assessed following lysostaphin treatment and viable intracellular bacterial recovery. Each experimental condition was performed in triplicate within each run, and all experiments were independently repeated three times to ensure reproducibility. The cells from technical replicate wells within each biological replicate were combined prior to RNA extraction, but the three independent biological replicates for each condition were processed and sequenced as separate libraries.

### RNA isolation and sequencing

Total RNA was extracted from both infected and control WT and IL10RA-KO MAC-T cells using the RNeasy Mini Kit (Qiagen, Germany) in accordance with the manufacturer’s instructions. To eliminate residual genomic DNA contamination, RNA samples were treated with DNase I (Fermentas, Waltham, MA, USA). RNA concentration and purity were determined spectrophotometrically by measuring absorbance ratios at 260/280 nm using a BioTek Cytation-5 system (Agilent). RNA integrity was assessed using the Agilent 2100 Bioanalyzer, and only samples with RNA integrity number (RIN) values greater than 7 were selected for library preparation. cDNA library construction and high-throughput sequencing were conducted at the Advanced Analysis Centre (AAC), University of Guelph, using the Illumina NextSeq 1000 platform.

### RNA-seq and differential expression analysis

Raw sequencing reads were processed and analyzed using CLC Genomics Workbench v25.0.1 (CLC Bio, Aarhus, Denmark). Initial quality-control assessments included evaluation of base quality scores, GC-content distribution, sequence-length distribution, nucleotide composition, ambiguous bases, and overrepresented sequences. Cleaned paired-end reads were aligned to the bovine reference genome (Bos taurus, ARS-UCD1.2), and mapping performance was assessed for each library. Differential expression testing was performed using mapped read counts as input to the generalized linear model (GLM)-based differential expression workflow implemented in CLC Genomics Workbench. RPKM values were calculated for expression-level reporting, visualization, and fold-change interpretation, but were not used as the input for statistical testing. Genes were considered significantly differentially expressed if they met the following thresholds: p-value < 0.001, false discovery rate (FDR) < 0.05, and absolute fold change ≥ 2. To further assess sample relationships and replicate consistency, principal component analysis (PCA) and/or hierarchical clustering was performed using normalized expression values across sequenced libraries.

To further compare transcriptional responses across conditions, overlapping DEG sets were visualized using Venn diagrams. Additional statistical evaluation of DEGs across treatment groups was performed using a Poisson-based model with significance criteria of FDR ≤ 0.05 and fold change ≥ 2.

### Gene ontology and pathway enrichment analysis

Functional annotation and enrichment analyses were conducted to interpret the biological significance of identified DEGs. Separate lists of upregulated and downregulated genes were uploaded to the SRplot online platform for Gene Ontology (GO) and Kyoto Encyclopedia of Genes and Genomes (KEGG) pathway analysis.

Enrichment analyses were performed using default parameters provided by the platform. Significantly enriched GO terms and KEGG pathways were identified based on platform-defined p-value thresholds. Results were visualized using bar plots displaying enrichment significance as −log_10_(p-value), along with the number of genes associated with each functional category, or pathway.

## Results

To evaluate whether IL10RA deletion induces an inherent, baseline transcriptional signature prior to infection, we first performed a direct comparative analysis between uninfected IL10RA-KO and uninfected WT control cells. At our established statistical thresholds (FDR < 0.05, |FC| ≥ 2), this comparison yielded zero differentially expressed genes (DEGs). This confirms that the knockout cell line does not possess a constitutively dysregulated transcriptome in its resting state, and that the subsequent transcriptional variations are strictly a consequence of staphylococcal challenge.

### Differentially expressed genes in response to *S. aureus* infection

To evaluate the transcriptional landscape of bovine mammary epithelial cells during *S. aureus* infection and the influence of IL10RA signaling, we performed RNA sequencing on WT and IL10RA-KO MAC-T cells challenged with either the *S. aureus* PS, or SCV. Across all libraries, 26,804 genes were identified. The DEGs were determined by comparing infected cells with their respective uninfected controls, and IL10RA-KO infected cells with WT infected counterparts (**Table 1**). The top 10 most significantly up- and down-regulated DEGs per group, ranked by fold change, are presented in **Table 2**.

**Table 1:** Expression profiles of differentially expressed (DE) genes across experimental groups.

Group	DE genes	Upregulated	Downregulated
WT-PS Infection vs WT Control	8	7	1
WT-SCV Infection vs WT Control	461	262	199
IL10RA-KO-PS Infection vs WT-PS Infection	619	290	329
IL10RA-KO-SCV Infection vs WT-SCV Infection	1297	661	636

WT, wild-type bovine mammary epithelial cell; PS, parental strain; SCV, small colony variant; KO, knockout; DE, differentially expressed. The total number of genes detected reflects the full transcriptome across all libraries before differential expression filtering.

**Table 2:** Top 10 upregulated and downregulated differentially expressed genes by experimental treatment.

Treatment	Regulation	Gene ID	FC	P-value	FDR
WT-PS Infected Vs WT Control	Up	ANKRD37	22.88128	4.94E-07	0.002727
		gene:ENSBTAG00000051139	18.6583	5.96E-06	0.015065
		STC1	14.32736	2.44E-07	0.00216
		DDIT4	10.42112	1.08E-05	0.023834
		gene:ENSBTAG00000050527	8.263139	6.17E-07	0.002727
		EGLN3	7.506958	3.65E-06	0.010764
		gene:ENSBTAG00000023274	3.910198	1.49E-06	0.005283
	Down	gene:ENSBTAG00000020685	-4.93634	4.18E-09	7.39E-05
WT-SCV Infected Vs WT Control	Up	ZNF536	39.72935	4.79E-15	3.72E-12
		MGC148328	25.41982	2.55E-06	0.000238
		U2_33	24.81769	0.000203	0.008737
		U1_29	16.49239	0.000235	0.009795
		gene:ENSBTAG00000032902	14.95874	1.24E-07	1.89E-05
		SLC14A1	14.34728	1.74E-44	1.55E-40
		PDCD1	14.20717	1.55E-07	2.25E-05
		POU2AF1	12.6369	1.25E-09	3.49E-07
		IFIT1	11.16344	4.05E-05	0.002501
		IRF8	10.58358	1.86E-15	1.58E-12
	Down	gene:ENSBTAG00000023823	-254.206	0.000855	0.026104
		TMPRSS11BNL	-229.545	0.001048	0.030508
		TGM3	-60.9612	5.42E-22	9.68E-19
		S100A8	-52.1201	2.71E-22	5.37E-19
		KRTDAP	-43.2342	1.32E-07	1.97E-05
		SBSN	-41.8128	3.3E-19	5.06E-16
		AMTN	-41.1097	3.57E-07	4.55E-05
		gene:ENSBTAG00000013507	-32.3967	2.4E-22	5.36E-19
		S100A9	-30.9775	4.84E-10	1.54E-07
		FAT3	-18.9472	2.26E-10	7.75E-08
IL10RA-KO-PS Infection vs WT-PS Infection	Up	CD79B	346.5058	0.00033	0.013075
		SAA3	131.8648	2.36E-06	0.000174
		gene:ENSBTAG00000052031	101.4946	7.26E-07	6.17E-05
		NOSTRIN	93.07905	2.72E-12	6.43E-10
		SLC1A3	83.0573	5.51E-09	7.9E-07
		HHIPL1	64.66057	3.32E-18	1.86E-15
		gene:ENSBTAG00000038770	58.65678	1.44E-05	0.000876
		WFDC2	57.59597	2.11E-50	1.89E-46
		RNF223	49.43071	9.81E-12	2.07E-09
		MYT1	46.80122	2.23E-24	2.5E-21
	Down	AMN	-4414.23	1.11E-06	9.12E-05
		PGAP4	-2924.56	3.99E-06	0.000279
		OSTN	-1805.84	1.57E-05	0.000931
		RFLNA	-1423.72	2.86E-05	0.00157
		PID1	-1095.66	5.46E-05	0.002784
		ZDHHC14	-896.465	2.16E-21	1.94E-18
		RIC3	-769.109	0.000127	0.005712
		FAT3	-704.543	0.000171	0.007311
		LY6E	-375.53	1.17E-28	1.61E-25
		GPC3	-330.418	0.000953	0.031043
IL10RA-KO-SCV Infection vs WT-SCV Infection	Up	CD79B	492.8668	0.0005	0.008407
		MYT1	218.6314	1.32E-57	3.4E-54
		gene:ENSBTAG00000038770	181.2548	0.003629	0.039518
		TNNC1	171.7033	0.003825	0.041099
		CD1D	166.6658	0.004142	0.043621
		TTC39A	104.34	5.53E-15	7.49E-13
		NOSTRIN	103.2356	4.09E-11	3.39E-09
		WFDC2	91.90114	5.3E-106	9.6E-102
		SLC1A3	83.89595	1.69E-06	6.17E-05
		ZNF23	77.877	4.21E-13	4.57E-11
	Down	AMN	-4465.28	6.17E-08	3.08E-06
		ZDHHC14	-4268.7	7.2E-08	3.54E-06
		PGAP4	-2607.6	4.1E-07	1.71E-05
		LY6E	-2355.91	2.36E-19	5.11E-17
		RIC3	-857.081	1.44E-05	0.000406
		RFLNA	-640.449	1.07E-13	1.22E-11
		5S_rRNA_278	-379.53	0.000504	0.008459
		CADPS2	-377.256	4.36E-18	8.02E-16
		WC1	-346.73	0.000184	0.003752
		U2_34	-239.697	0.000992	0.014542

WT, wild type MAC-T cell; FC, fold change; Up, upregulated genes; Down, downregulated genes; FDR, false discovery rate (Benjamini–Hochberg adjusted p-value); MAC-T, bovine mammary alveolar epithelial cell line; PS, parental strain; SCV, small colony variant; KO, knockout.

In the WT MAC-T cells, the PS infection elicited a modest transcriptional response of only 8 DEGs (7 up-regulated, 1 down-regulated; **Table 1**), with the most highly induced genes being *ANKRD37*, *STC1* and *DDIT4*, associated with hypoxia-inducible factor signaling and cellular stress adaptation. In contrast, the SCV infection elicited a robust transcriptional response of 461 DEGs (262 up-regulated, 199 down-regulated), a 57.6-fold more robust response over PS infection. The most strongly down-regulated host genes in SCV-infected WT cells included *TGM3*, *S100A8*, *KRTDAP* and *SBSN*, several of which are implicated in epithelial barrier integrity, keratinocyte differentiation, and antimicrobial defense (**Table 2**). Critically, there was next to no transcriptional overlap between the WT-PS and WT-SCV sets of DEGs (**Figures 1A, B**).

**Figure 1:**
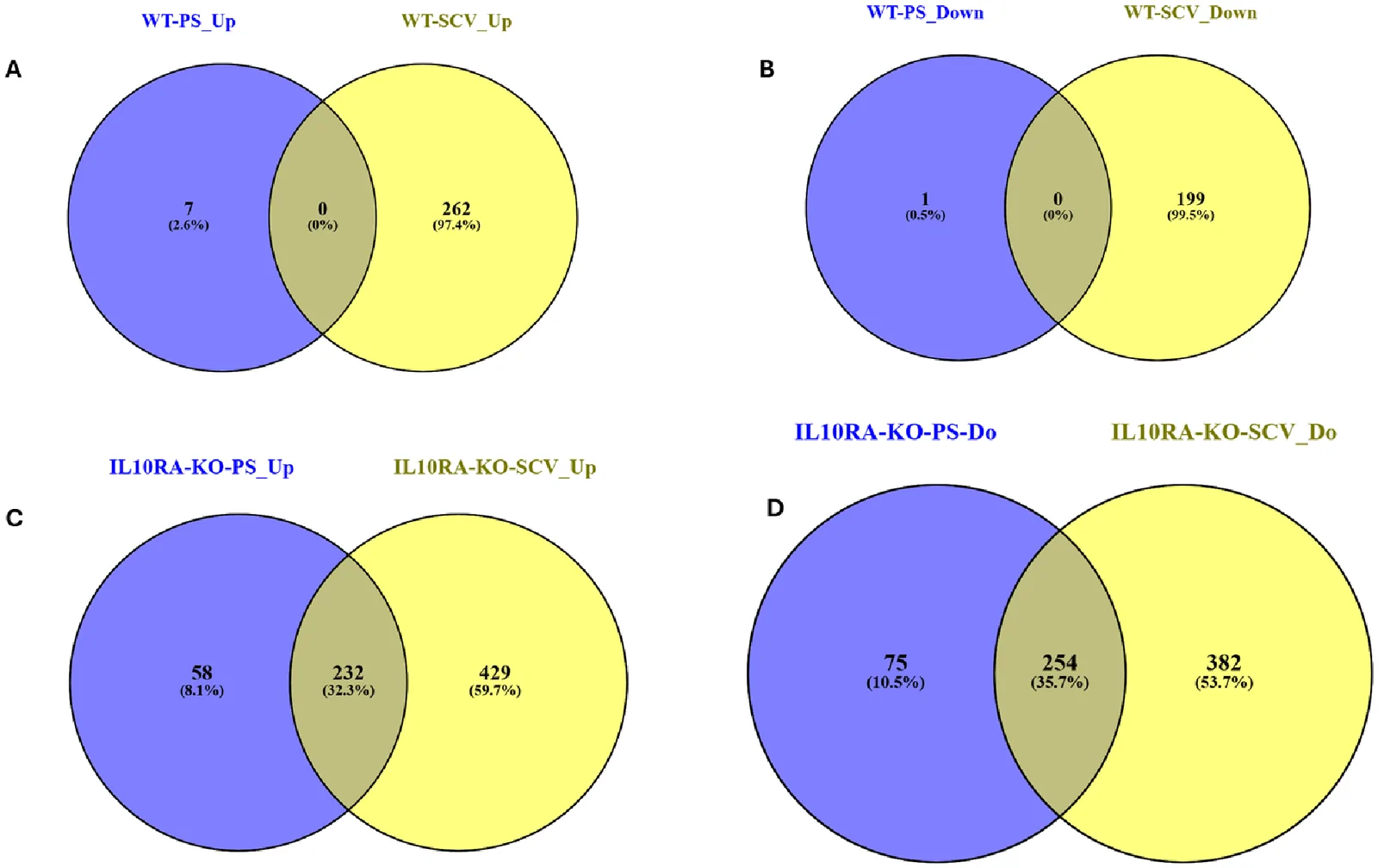
Venn diagrams illustrating the overlap of differentially expressed genes across experimental groups. **(A)** Overlap of upregulated DEGs between wildtype MAC-T cells infected with *S. aureus* parental strain (WT-PS) and small colony variant (WT-SCV). **(B)** Overlap of downregulated DEGs between wildtype MAC-T cells infected with *S. aureus* parental strain (WT-PS) and small colony variant (WT-SCV). **(C)** Overlap of upregulated DEGs between IL10RA-KO MAC-T cells infected with parental strain (IL10RA-KO-PS_UP) and small colony variant (IL10RA-KO-CSV_UP). **(D)** Overlap of downregulated DEGs between IL10RA-KO MAC-T cells infected with parental strain (IL10RA-KO-PS_Do) and small colony variant (IL10RA-KO-CSV_Do). Numbers within each region indicate the count and percentage of genes unique to each condition or shared between groups. DEGS, differentially expressed genes; MAC-T, bovine mammary alveolar epithelial cell line; WT, wild type; PS, parental strain; SCV, small colony variant; IL10RAKO, Interleukin 10 receptor alpha knockout.

IL10RA disruption substantially amplified transcriptional dysregulation under both infection conditions. The IL10RA-KO cells infected with PS for example, yielded 619 DEGs (77.4-fold increase over WT-PS), while the IL10RA-KO cells infected with SCV yielded 1,297 DEGs (2.8-fold increase over WT-SCV; **Table 1**). The fold-change magnitudes in both KO groups far exceeded those of the corresponding WT conditions. The most dramatic up-regulated gene in both IL10RA-KO infection groups was *CD79B* (FC = 346.51 in IL10RA-KO-PS; FC = 492.87 in IL10RA-KO-SCV), and the most extreme correspondingly down-regulated genes were *AMN* (FC = −4,414 and −4,465, respectively) and *PGAP4* (**Table 2**). The recurrence of *CD79B*, *WFDC2*, *MYT1*, *NOSTRIN*, *AMN* and *PGAP4* among the top DEGs in both IL10RA-KO conditions point to a core set of IL10RA-regulated genes central to mammary epithelial homeostasis. The up-regulated and down-regulated genes were approximately equally balanced in both IL10RA-KO comparisons ~47–51% down-regulated).

Comparison of DEG sets between the two IL10RA-KO conditions revealed that 232 of 661 up-regulated genes (32.3%) and 254 of 711 down-regulated genes (35.7%) were shared, with the majority of condition-specific DEGs unique to the SCV condition (**Figures 1C, D**).

### Functional enrichment of up-regulated DEGs in WT-PS versus WT-control groups

GO enrichment of upregulated DEGs in the WT cells infected with the PS identified stress-response and apoptosis-related processes as the most significantly enriched terms, including cell migration, cell motility, regulation of anion transport, and intrinsic apoptotic signaling pathway by p53 class mediator (**Figure 2**).

**Figure 2:**
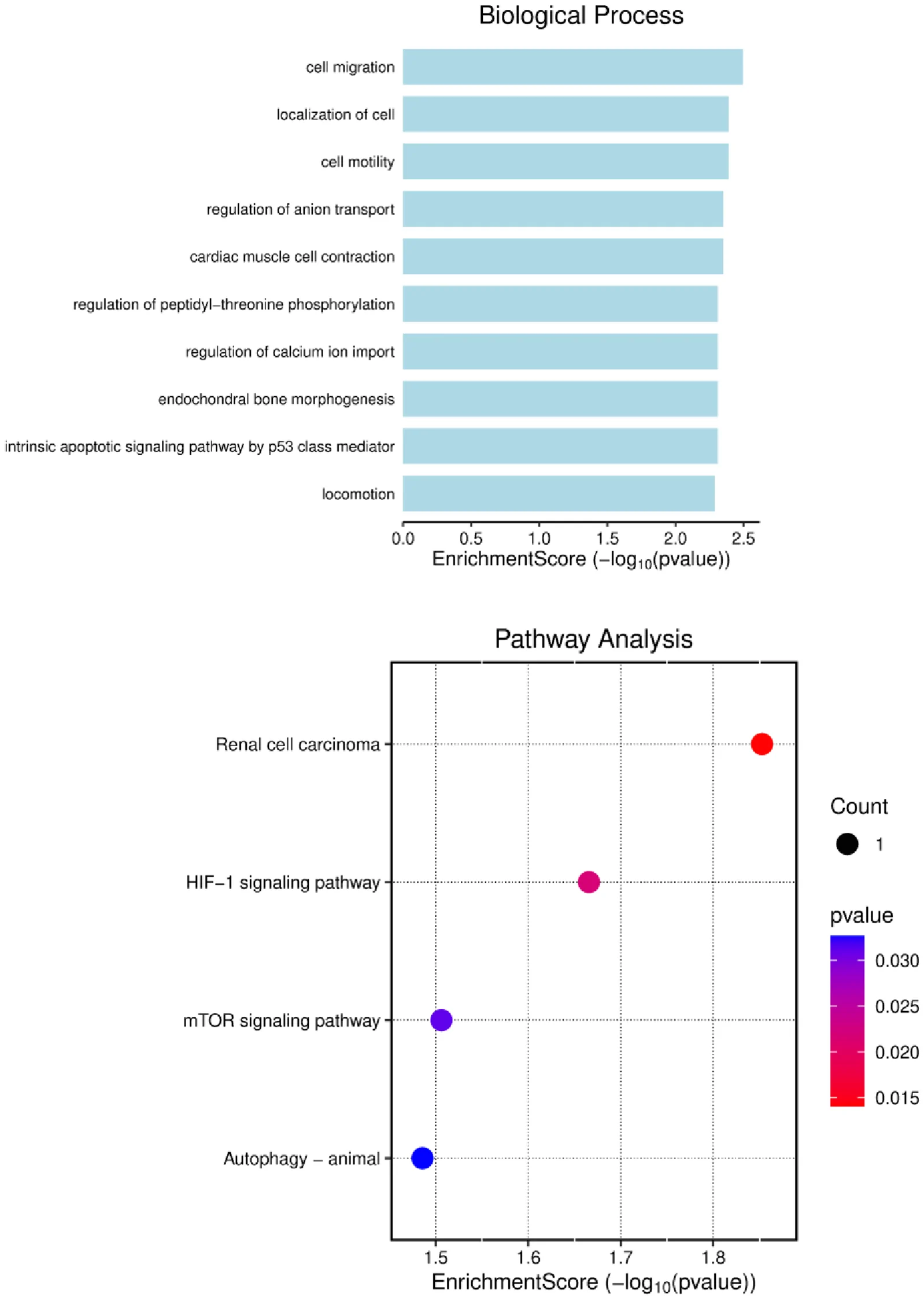
Functional enrichment analysis of upregulated differentially expressed genes in wild-type MAC-T cells infected with *Staphylococcus aureus* parental strain (WT-PS) compared with uninfected controls (WT-Control). (Top) Gene Ontology (GO) biological process enrichment dot plot showing the top 10 enriched terms. Dot size represents the number of genes mapped to each term; dot color indicates the p-value. The x-axis represents the enrichment score expressed as −log_10_(p-value). (Bottom) KEGG pathway enrichment bar chart showing the top 10 enriched pathways ranked by −log_10_(p-value). Only upregulated DEGs were observed in this comparison.

### Functional enrichment of DEGs in WT-SCV versus WT-control groups

Up-regulated DEGs in SCV-infected WT cells were enriched predominantly in lipid and sterol metabolic processes, with cholesterol biosynthesis, sterol metabolic process, and steroid biosynthesis as the top GO and KEGG terms (**Figure 3A**). Down-regulated DEGs were enriched in processes related to cellular architecture, adhesion, and extracellular matrix organization, with Cornified envelope formation and Hippo signaling pathway as the leading KEGG pathways, followed by Complement and coagulation cascades and TGF-β signaling (**Figure 3B**).

**Figure 3:**
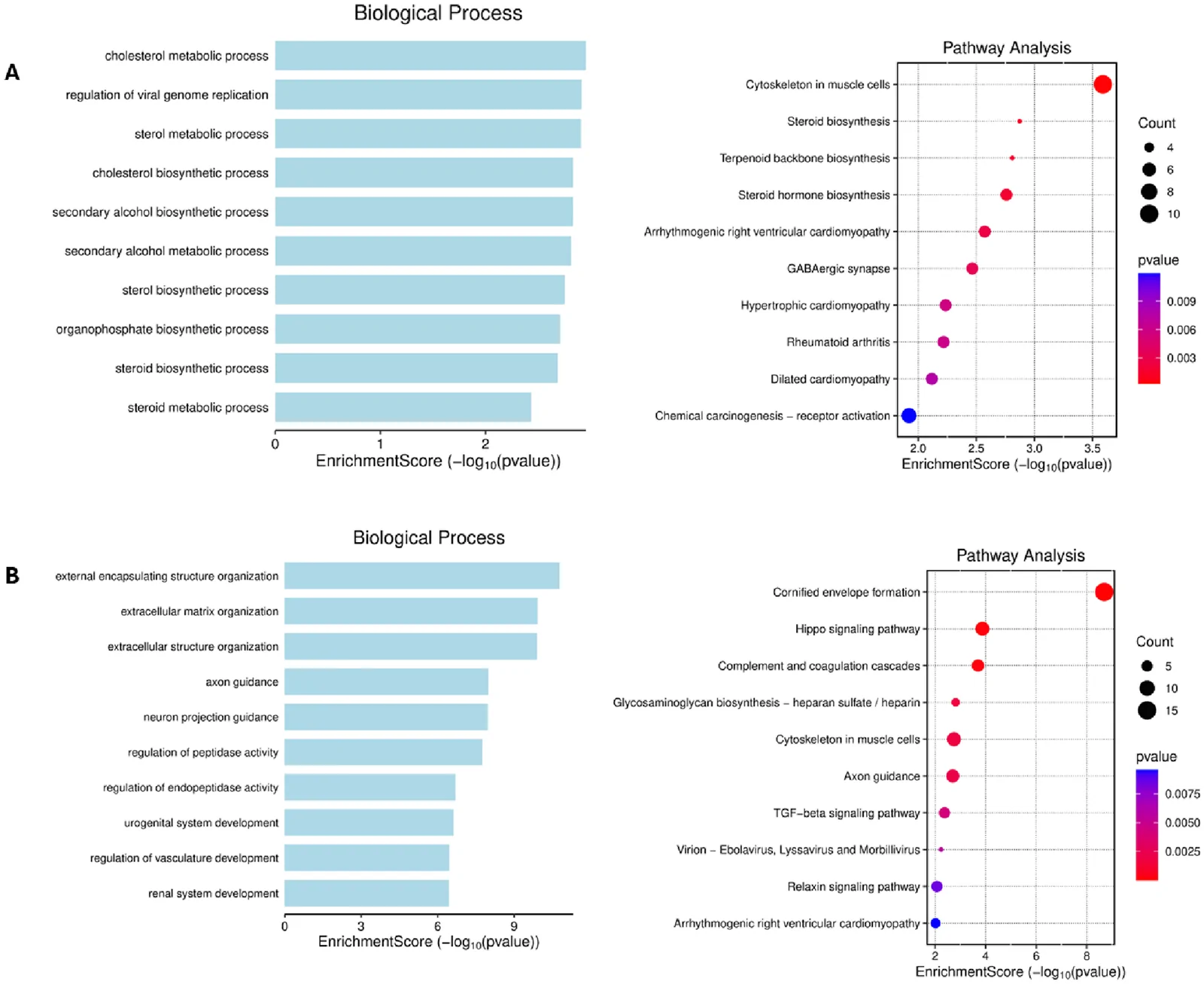
Functional enrichment analysis of differentially expressed genes in wild-type MAC-T cells infected with the *Staphylococcus aureus* small colony variant (WT-SCV) compared with uninfected controls (WT-Control). **(A)** Upregulated DEGs: (left) GO biological process enrichment dot plot showing the top 10 enriched terms, with dot size representing gene count and dot color indicating p-value; (right) KEGG pathway enrichment bar chart showing the top 10 enriched pathways. **(B)** Downregulated DEGs: (left) GO biological process enrichment dot plot; (right) KEGG pathway enrichment bar chart.

### Functional enrichment of DEGs in IL10RA-KO-PS versus WT-PS groups

Upregulated DEGs in IL10RA-KO cells following PS infection were enriched in immune activation and differentiation pathways (**Figure 4A**). The top KEGG pathways included Cytokine–cytokine receptor interaction, Allograft rejection, TGF-β signaling, and Antigen processing and presentation. Down-regulated DEGs in the same comparison were markedly enriched in lipid metabolic and transport processes, with steroid biosynthesis, cholesterol biosynthetic process, PPAR signaling, and Terpenoid backbone biosynthesis as the leading KEGG pathways (**Figure 4B**).

**Figure 4:**
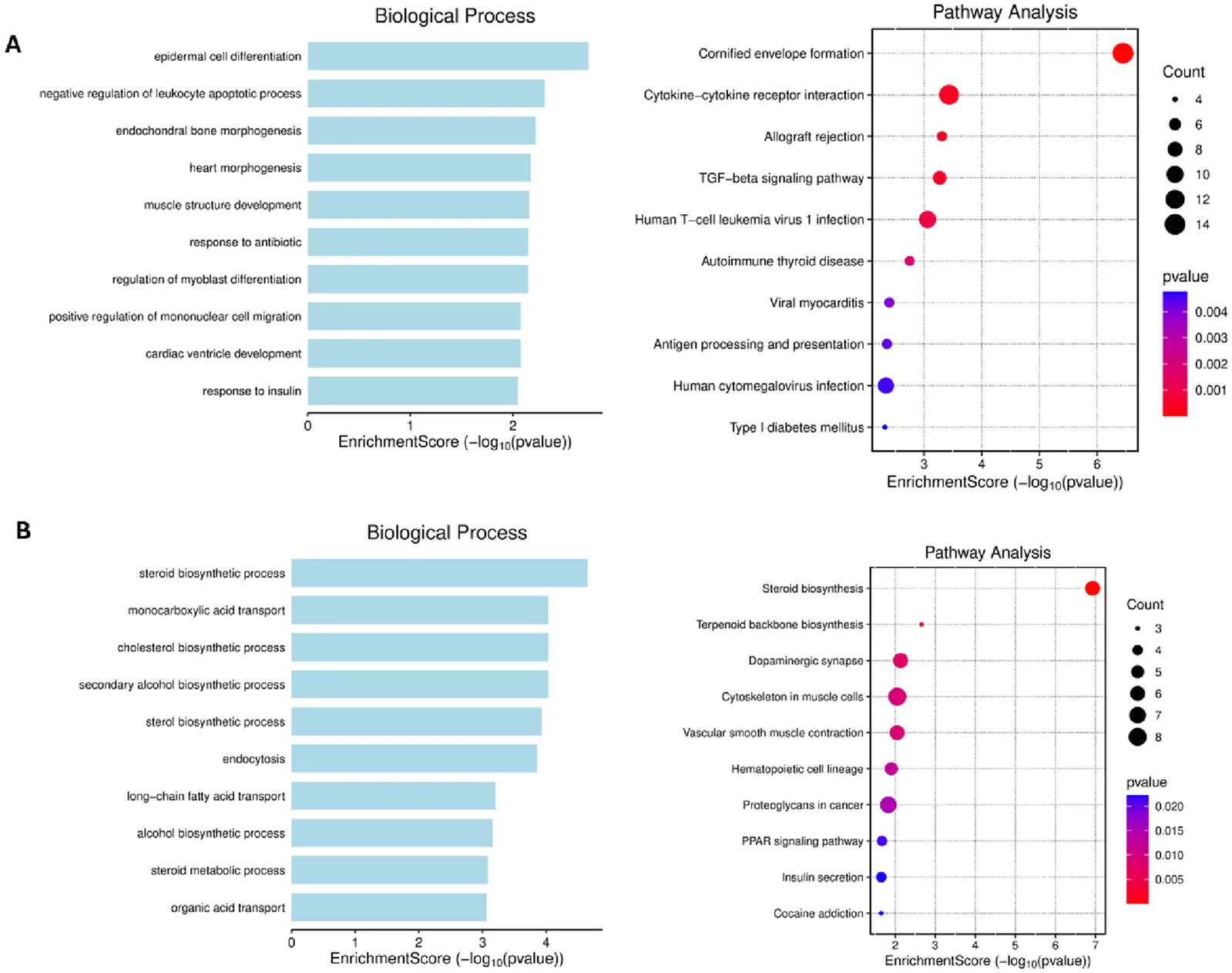
Functional enrichment analysis of differentially expressed genes comparing IL10RA-knockout MAC-T cells infected with the *Staphylococcus aureus* parental strain (IL10RA-KO-PS) to wild-type MAC-T cells infected with the parental strain (WT-PS). **(A)** Upregulated DEGs: (left) GO biological process enrichment dot plot showing the top 10 enriched terms, with dot size representing gene count and dot color indicating p-value; (right) KEGG pathway enrichment bar chart showing the top 10 enriched pathways. **(B)** Downregulated DEGs: (left) GO biological process enrichment dot plot; (right) KEGG pathway enrichment bar chart.

### Functional enrichment of DEGs in IL10RA-KO-SCV versus WT-SCV groups

Up-regulated DEGs in IL10RA-KO cells infected with the SCV were enriched in pro-apoptotic biological processes (positive regulation of cell death, positive regulation of apoptotic process) and pro-inflammatory signaling pathways (**Figure 5A**). The leading KEGG pathways were TNF signaling, IL-17 signaling, and Cytokine–cytokine receptor interaction, alongside Leukocyte transendothelial migration. Down-regulated DEGs in IL10RA-KO-SCV cells were enriched in endocytosis, fatty acid transport, and organic acid transport processes, with Phagosome and Steroid biosynthesis among the top KEGG pathways (**Figure 5B**).

**Figure 5:**
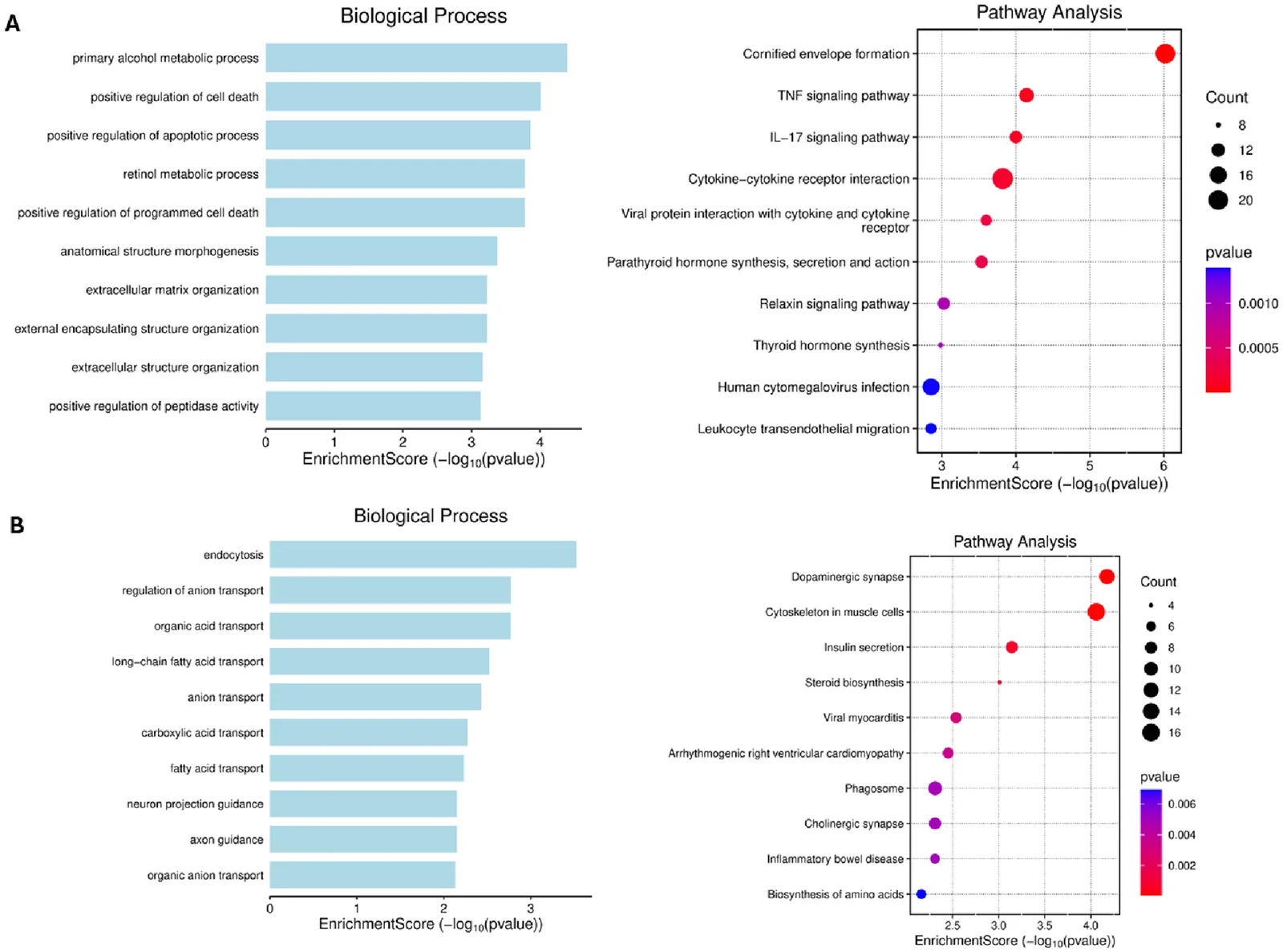
Functional enrichment analysis of differentially expressed genes comparing IL10RA-knockout MAC-T cells infected with the *Staphylococcus aureus* small colony variant (IL10RA-KO-SCV) to wild-type MAC-T cells infected with the small colony variant (WT-SCV). **(A)** Upregulated DEGs: (left) GO biological process enrichment dot plot showing the top 10 enriched terms, with dot size representing gene count and dot color indicating p-value; (right) KEGG pathway enrichment bar chart showing the top 10 enriched pathways. **(B)** Downregulated DEGs: (left) GO biological process enrichment dot plot; (right) KEGG pathway enrichment bar chart.

## Discussion

The present study provides the first comprehensive transcriptomic characterization of IL10RA-dependent host responses in bovine MECs challenged with PS and SCV stains of *S. aureus*. Using IL10RA-KO and WT MAC-T cells and RNA sequencing, we identified four key findings: (i) the PS and SCV elicit profoundly divergent transcriptional programs in WT MAC-T cells; (ii) IL10RA disruption dramatically amplifies transcriptional dysregulation; (iii) a moderate core of IL10RA-regulated genes (~32-36%) is shared across both PS and SCV infection conditions; and (iv) IL10RA governs pro-inflammatory cytokine signaling, lipid metabolic, apoptotic, and structural processes. Together, these findings implicate IL10RA as a critical modulator of epithelial transcriptional balance during intracellular *S. aureus* challenge.

### Divergent transcriptional responses of MAC-T cells to PS and SCV *S. aureus* infection

A striking feature of the present study was the near-complete divergence in transcriptional responses of WT MAC-T cells to PS and SCV infection. No DEGs were shared between the WT-PS (8 DEGs) and WT-SCV (461 DEGs) groups, indicating that these isogenic strains activate fundamentally distinct molecular programs in the same host-cell type. The modest response to PS infection, comprising 7 up-regulated and 1 down-regulated gene, is consistent with the well-documented capacity of *S. aureus* to actively dampen early innate immune activation in mammary epithelial cells. Günther et al. (2017) for example, demonstrated that viable *S. aureus*, unlike *Escherichia coli*, fail to robustly induce NF-κB activation, IL-8, or TNF-α in the bovine udder, despite heat-killed preparations being capable of equivalently activating bovine *TLR2* and *TLR4*. This immune evasion has been attributed, in part, to rapid intracellular sequestration of the bacterium, thereby limiting extracellular PRR engagement (Günther et al., 2009). The predominantly TLR2-mediated rather than TLR4-mediated recognition of *S. aureus* lipoteichoic acid (Schwandner et al., 1999), may further explain the attenuated transcriptional response of MAC-T cells to the PS compared with that elicited by Gram-negative pathogens through the LPS–TLR4 axis (Gilbert et al., 2013).

The upregulated DEGs in PS-infected WT cells were enriched in HIF-1 and mTOR signaling pathways, and included the hypoxia-inducible genes *ANKRD37*, *STC1*, *DDIT4*, and *EGLN3*. The induction of these HIF-1α transcriptional targets is consistent with the metabolic stress and hypoxic microenvironment created by intracellular bacterial replication. *DDIT4* (also known as REDD1) functions as an mTORC1 suppressor under hypoxic and energy-stress conditions (Brugarolas et al., 2004), while *ANKRD37* and *EGLN3* are canonical HIF-1α targets involved in the cellular hypoxia-response program (Pescador et al., 2005). These findings align with Zhao et al. (2025a), who noted consistent upregulation of HIF-1 pathway components, including *ANKRD37* and *EGLN3*, in *S. aureus*-infected MAC-T cells, reinforcing the centrality of hypoxic stress adaptation in early host responses to staphylococcal infection. The enrichment of autophagy and apoptotic pathways (renal cell carcinoma, mTOR signaling, autophagy–animal) at the KEGG level is consistent with intrinsic apoptotic cascades following *S. aureus* invasion, as demonstrated by Bayles et al. (1998), who showed that intracellular *S. aureus* escapes the endosome and triggers mitochondria-dependent apoptosis in epithelial cells.

In contrast, SCV infection provoked a markedly broader transcriptional response encompassing 461 DEGs. Among the most strongly up-regulated transcripts during SCV infection were *ZNF536, PDCD1, SLC14A1* and *IRF8*. The induction of PDCD1, which encodes the immune checkpoint receptor PD-1 (Sharpe and Pauken, 2018), is noteworthy as a transcriptional finding; however, because RNA-seq measures transcript abundance rather than protein expression, this result should not be interpreted as evidence of increased PD-1 protein without further validation. Future studies using immunoblotting or immunofluorescence staining will be required to determine whether PDCD1 transcript induction translates into increased PD-1 protein expression in SCV-infected MAC-T cells. *IRF8* encodes Interferon Regulatory Factor 8, a transcription factor critical for macrophage and dendritic cell differentiation and innate immune gene expression; its upregulation may reflect an attempt by the infected epithelium to recruit or mimic myeloid-type immune defense programs during the extensive intracellular colonization associated with SCV persistence (Tamura et al., 2005).

The most markedly down-regulated genes in SCV-infected WT cells, including *TGM3*, *S100A8*, *S100A9*, *KRTDAP* and *SBSN*, are strongly associated with epithelial barrier integrity, keratinocyte differentiation, and antimicrobial defense. Down-regulation of *S100A8* and *S100A9* is particularly significant; these calcium-binding pro-inflammatory proteins are major activators of NF-κB signaling and are strongly upregulated in the bovine mammary gland during acute staphylococcal mastitis (Wang et al., 2021). Their suppression during SCV infection is consistent with the concept that SCVs actively dampen pro-inflammatory signaling pathways to facilitate intracellular persistence, a strategy extensively documented in airway epithelial models where SCV intracellular infection induced up-regulation of only *TLR2* with minimal pro-inflammatory cytokine induction (Ou et al., 2016). Down-regulation of *TGM3* (transglutaminase 3) and *KRTDAP* (keratinocyte differentiation-associated protein) further indicates SCV-mediated disruption of the cornified envelope and keratinocyte differentiation programs, processes critical for maintaining the structural integrity of mucosal epithelial barriers (Simon et al., 2009).

At the functional level, GO and KEGG enrichment of up-regulated genes in WT-SCV cells revealed a dominant lipid and sterol metabolic signature, including enrichment in cholesterol biosynthesis, steroid biosynthesis, and terpenoid backbone biosynthesis pathways (**Figure 3A**). This metabolic reprogramming mirrors observations reported in other *S. aureus* infection contexts. Bivona et al. (2024) demonstrated that SCV formation in macrophages was associated with metabolic adaptation, and Kohler et al. (2003) showed that hemB mutant SCVs preferentially use fermentative metabolic pathways to generate ATP in acidic phagolysosomal environments. Enrichment of host cholesterol and sterol biosynthesis during SCV infection likely reflects increased demand for lipid membrane components as the host cell accommodates enlarging intracellular vacuolar compartments around persisting SCVs, consistent with the transmission electron microscopy observations of Atalla et al. (2010a) documenting large vacuolar structures containing SCV Heba3231 in MAC-T cells. Concomitantly, the down-regulation of Hippo, Wnt, complement and coagulation cascade, and cornified envelope pathways (**Figure 3B**), suggests that SCV-mediated intracellular persistence is accompanied by broad perturbation of the epithelial structural integrity program, a finding consistent with recent transcriptomic studies showing that *S. aureus* infection of MAC-T cells preferentially perturbs structural and metabolic gene programs while suppressing TLR–NF-κB-driven inflammatory responses (Zhao et al., 2025a).

### IL10RA disruption dramatically amplifies transcriptional dysregulation during staphylococcal infection

One of the most compelling findings of this study was the dramatic amplification of transcriptional dysregulation in IL10RA-KO cells; the IL10RA-KO cells infected with the PS yielded 619 DEGs (a 77.4-fold increase over WT-PS), and the IL10RA-KO cells infected with SCV yielded 1,297 DEGs (a 2.8-fold increase over WT-SCV). This finding provides the first genome-wide evidence that IL10RA exerts a broad transcriptional regulatory function in MAC-T cells during *S. aureus* infection, far exceeding its classical role as the anti-inflammatory interleukin-10 signaling receptor.

Interleukin-10 (IL-10) is the principal anti-inflammatory cytokine of the mammary immune environment, signaling exclusively through the heterodimeric IL-10 receptor composed of IL10RA and IL10RB subunits (Ouyang et al., 2011). Upon IL-10 binding, IL10RA activates STAT3 phosphorylation, which suppresses pro-inflammatory NF-κB signaling, reduces the production of TNF-α, IL-6, IL-12, and IL-17, and promotes immunosuppressive and anti-inflammatory transcriptional programs (Murray, 2006). In the bovine mammary gland, IL-10 plays a well-recognized role in limiting inflammatory tissue damage during mastitis, and elevated IL-10 expression has been associated with both resolution of acute mastitis and, paradoxically, with increased susceptibility to persistent infection by attenuating the bactericidal inflammatory response (Ibeagha-Awemu et al., 2008). The dramatic transcriptional amplification observed upon IL10RA loss, therefore, indicates that under normal conditions, IL10RA-mediated signaling actively restrains a large proportion of the host transcriptional response to *S. aureus* infection, functioning as a key brake on both inflammatory and structural gene programs in MAC-T cells.

The enrichment of cytokine–cytokine receptor interaction, TNF signaling, IL-17 signaling, allograft rejection, antigen processing and presentation, and TGF-β signaling pathways in the up-regulated DEGs of IL10RA-KO cells infected with either PS, or SCV (**Figures 4A**, **5A**) is consistent with the classical immunosuppressive role of the IL-10/IL10RA axis. Loss of IL10RA-mediated STAT3 activation would be expected to derepress NF-κB and MAPK signaling, releasing a broad range of pro-inflammatory transcriptional programs. The enrichment of TNF and IL-17 signaling pathways specifically is particularly relevant to bovine mastitis pathogenesis; TNF-α is a central mediator of mammary gland inflammation and tissue damage during acute mastitis (Sordillo et al., 1997), while IL-17 signaling has been shown to coordinate neutrophil recruitment and antimicrobial responses during early *S. aureus* infection of the bovine udder (Wang et al., 2021). The concurrent upregulation of antigen processing and presentation, viral myocarditis, and autoimmune thyroid disease pathways suggests a broader immunological dysregulation triggered by the removal of IL-10-mediated immune tolerance signals, analogous to the phenotypes observed in mice with targeted IL10RA deletion, which develop severe spontaneous colitis due to unrestrained mucosal immune activation (Spencer et al., 1998).

Identification of *CD79B* as one of the most highly up-regulated transcripts in both IL10RA-KO infection conditions was unexpected and intriguing. *CD79B* encodes Ig-β, a component of the B-cell receptor signaling complex that is canonically expressed in B lymphocytes. While its transcript-level induction in MAC-T cells suggests a possible non-canonical epithelial response associated with IL10RA disruption, this finding should be interpreted cautiously because protein-level expression was not assessed in the present study. Immunoblotting or immunofluorescence staining will be required in future studies to confirm whether the observed CD79B transcriptional increase results in detectable CD79B protein expression in IL10RA-KO MAC-T cells. Similarly, *WFDC2* (WAP Four-Disulfide Core Domain Protein 2, also known as HE4) was among the top up-regulated genes in both IL10RA-KO conditions (FC = 57.60 in IL10RA-KO-PS; FC = 91.90 in IL10RA-KO-SCV). *WFDC2* encodes an antiproteinase with innate immune functions at mucosal surfaces and has been implicated in epithelial defense (Bingle and Vyakarnam, 2008). Its recurrent up-regulation in IL10RA-KO MAC-T cells mirrors its pattern in *TLR4*-KO MAC-T cells (Shandilya et al., 2021), suggesting that *WFDC2* induction may represent a broadly conserved compensatory antimicrobial response activated when primary innate immune receptor-mediated signaling, whether TLR4 or IL10RA, is disrupted.

The recurrence of *MYT1*, *NOSTRIN*, and *SLC1A3* among the top up-regulated genes in both IL10RA-KO infection conditions warrants further investigation. NOSTRIN (nitric oxide synthase trafficker) is a regulator of endothelial nitric oxide synthase (eNOS) localization and activity (Schilling et al., 2006), and its marked upregulation in IL10RA-KO cells may indicate dysregulated nitric oxide production, a mechanism used by host cells to kill intracellular bacteria. *SLC1A3* encodes the glutamate transporter GLAST, and its upregulation may reflect altered metabolic demands or amino acid availability in the *S. aureus*-infected IL10RA-deficient epithelial environment (Bhatt et al., 2020).

### Core IL10RA-regulated gene programs are partially shared across PS and SCV infection

A notable feature of the transcriptional data was the moderate overlap between IL10RA-KO-PS and IL10RA-KO-SCV conditions. Approximately 32.3% of up-regulated genes and 35.7% of down-regulated genes were shared between the two KO infection conditions (**Figures 1C, D**). This degree of overlap, while meaningful in identifying a core IL10RA-regulated gene set, is substantially lower than the ~57% overlap reported between *TLR4*-KO conditions in an otherwise analogous experimental system (Shandilya et al., 2021). This difference suggests that, while both IL10RA and TLR4 regulate gene programs shared across *S. aureus* strains, the IL10RA-regulated transcriptional response is more strongly shaped by the specific characteristics of the infecting phenotype, particularly the SCV. This is biologically plausible, given that IL-10 signaling is primarily driven by the magnitude and nature of the preceding inflammatory response, rather than by direct pathogen recognition, and the distinct inflammatory environments created by PS versus SCV infection would, therefore, produce quantitatively and qualitatively different IL-10 signaling demands (Ouyang et al., 2011).

The severely down-regulated genes in both IL10RA-KO infection conditions, including *AMN*, *PGAP4*, *ZDHHC14*, *LY6E* and *RIC3*, encode proteins with roles in membrane lipid remodeling, GPI anchor biosynthesis, and cellular homeostasis. *AMN* encodes amnionless, a co-receptor for megalin/LRP2 involved in vitamin B12 and cubilin-mediated endocytosis (Fyfe et al., 2004), while PGAP4 is involved in GPI anchor remodeling in the endoplasmic reticulum (Murakami et al., 2012). The extreme suppression of these membrane homeostasis genes (FC = −4,414 to −4,465 for *AMN*; FC = −2,925 to −2,608 for *PGAP4*) upon IL10RA loss points to a functional role for IL10RA signaling in maintaining membrane trafficking and endosomal processing programs in MAC-T cells, processes that may be relevant to phagolysosomal maturation and intracellular bacterial clearance. *LY6E* (lymphocyte antigen 6E) encodes a GPI-anchored protein implicated in viral and bacterial membrane fusion events (Yu et al., 2019); its down-regulation in IL10RA-KO cells may indicate impaired innate restriction of intracellular pathogens. The down-regulation of *ZDHHC14*, a DHHC-palmitoyl acyltransferase involved in protein S-palmitoylation and Ras signaling regulation (Fukata et al., 2004), further suggests that IL10RA is required to maintain the post-translational lipid modification machinery necessary for normal epithelial signaling and host defense.

The predominance of SCV-specific DEGs in both the up-regulated (59.7%) and down-regulated (53.7%) fractions of IL10RA-KO conditions (**Figures 1C, D**) indicates that IL10RA exerts particularly extensive regulatory control over the host response to SCV infection. The SCV engages a fundamentally different host–pathogen interaction than PS. Its capacity for prolonged intracellular residence within intact vacuoles (Atalla et al., 2010a), resistance to phagolysosomal acidification (Schröder et al., 2006), and suppression of NF-κB and NLRP3 inflammasome activation (Swindle et al., 2016; Zhao et al., 2025b), leads to a chronic low-grade intracellular infection that generates a qualitatively distinct IL-10 signaling demand compared with the more transient PS infection. This extended temporal window for IL10RA-mediated regulation would be expected to expand the breadth of IL10RA-dependent transcriptional control during SCV infection, consistent with the higher absolute DEG counts observed in the IL10RA-KO-SCV condition.

Because PS 3231 and SCV Heba3231 differ markedly in intracellular kinetics, the single 3.5 ah sampling point should be interpreted as capturing the acute/initiating transcriptional response to early intracellular infection rather than the full trajectory of chronic SCV persistence. This common early time point was intentionally used to compare genetically matched strains under matched exposure conditions before their infection trajectories diverge substantially, with PS becoming non-recoverable by 24 ah and SCV persisting intracellularly for up to 96 ah (Atalla et al., 2010a). A longitudinal RNA-seq time course, for example at 3.5, 24, 48, and 96 ah post-infection, will be important in future work to define how the host transcriptional response evolves as SCV persistence is established and maintained.

However, because the present study was designed as a transcriptomic analysis, these data do not establish whether IL10RA functionally promotes or restricts intracellular SCV persistence. The observed IL10RA-dependent changes identify host pathways that may influence putative intracellular bacterial handling pathways, but direct functional assays, such as gentamicin protection assays with viable intracellular CFU enumeration over time, will be required to determine whether these transcriptional differences alter SCV survival in MAC-T cells.

### IL10RA governs pro-inflammatory cytokine signaling and apoptotic coordination during SCV infection

The enrichment of pro-apoptotic biological processes, including positive regulation of cell death, positive regulation of apoptotic process, and positive regulation of programmed cell death, in up-regulated DEGs of IL10RA-KO cells infected with SCV (**Figure 5A**) is particularly noteworthy, given that SCV infection of WT MAC-T cells does not typically elicit strong apoptotic signaling. Atalla et al. (2010a) demonstrated that the SCV Heba3231 caused minimal cytopathic effects, remaining within intact vacuoles for up to 96 hours, whereas, the PS 3231 escaped the phagosome and caused rapid cell death by 24 hours. The emergence of apoptotic gene expression in IL10RA-KO cells specifically during SCV infection, therefore, suggests that IL-10/IL10RA signaling normally functions to suppress SCV-triggered apoptotic programs, potentially as a mechanism to preserve the intracellular niche that facilitates chronic persistence. This is consistent with the known anti-apoptotic functions of IL-10 in epithelial cells; IL-10 has been shown to inhibit Fas-mediated and TNF-induced apoptosis through STAT3-dependent up-regulation of anti-apoptotic Bcl-2 family members (Bhattacharya et al., 2005). Loss of this IL10RA-mediated pro-survival signaling during SCV infection would be expected to unmask latent apoptotic programs.

The enrichment of the TNF signaling pathway (**Figure 5A**) among up-regulated DEGs in IL10RA-KO-SCV cells is mechanistically coherent with this apoptotic signature. TNF-α is a potent inducer of both NF-κB-mediated inflammation and caspase-8-dependent extrinsic apoptosis; IL-10 normally counteracts TNF signaling through STAT3-mediated transcriptional suppression of TNF-α production and TNFR1 expression (Murray, 2006). Derepression of TNF signaling in the absence of IL10RA would thus be predicted to simultaneously amplify inflammatory gene expression and promote apoptotic cell death, a dual effect consistent with the concurrent enrichment of pro-inflammatory cytokine pathways and pro-apoptotic biological process terms observed in IL10RA-KO-SCV cells. The enrichment of Leukocyte transendothelial migration, IL-17 signaling, and Cytokine–cytokine receptor interaction further supports a scenario in which IL10RA loss triggers a broadly dysregulated, hyper-inflammatory epithelial transcriptional state during SCV infection.

Conversely, the downregulation of the phagosome pathway among IL10RA-KO-SCV downregulated DEGs (**Figure 5B**) is consistent with impaired intracellular bacterial killing. Phagosome maturation and acidification are essential for lysosomal bacterial clearance, and SCVs are known to subvert this process to establish persistent intracellular niches (Schröder et al., 2006). The finding that IL10RA loss reduces the expression of phagosome-associated genes suggests that IL10RA signaling may contribute to transcriptional regulation of intracellular bacterial-handling pathways during SCV infection. However, whether these transcriptomic changes enhance, reduce, or have no measurable effect on viable intracellular SCV persistence cannot be determined from RNA-seq alone and will require direct intracellular bacterial survival assays. This would be analogous to the immunopathological scenario in chronic inflammatory bowel disease, where IL-10/IL10RA deficiency leads to uncontrolled mucosal inflammation that fails to clear luminal bacteria (Shouval et al., 2014).

### IL10RA regulates lipid metabolic and structural programs in mammary epithelial cells

A consistent theme across multiple comparisons was the involvement of lipid biosynthesis and steroid metabolic pathways. In WT-SCV cells, lipid and sterol biosynthesis pathways were prominently upregulated (**Figure 3A**), while in IL10RA-KO-PS cells, these same pathways were markedly downregulated (**Figure 4B**). This reciprocal pattern suggests that IL10RA signaling normally promotes expression of lipid metabolic genes during PS infection, and that the upregulation of lipid biosynthesis observed in WT-SCV cells may be partially independent of IL10RA signaling. The suppression of steroid biosynthesis, PPAR signaling, vascular smooth muscle contraction, and hematopoietic cell lineage pathways in IL10RA-KO-PS downregulated DEGs (**Figure 4B**) indicates a broad disruption of lipid-dependent signaling programs. PPAR signaling is particularly relevant: PPARγ activation has anti-inflammatory effects in mammary epithelial cells and is known to suppress NF-κB-driven mastitis-associated gene expression (Tian et al., 2013). The concurrent loss of PPARγ pathway activity and gain of TNF/IL-17 signaling in IL10RA-KO cells thus creates a pro-inflammatory transcriptional environment that mirrors the immunopathology of severe acute mastitis.

The downregulation of structural and extracellular matrix pathways, including cytoskeleton in muscle cells, cornified envelope formation, complement and coagulation cascades, ECM–receptor interaction, and axon guidance, in both IL10RA-KO infection conditions is consistent with a broad role for IL10RA in maintaining epithelial structural homeostasis. Complement and coagulation cascade suppression is particularly noteworthy in the context of bovine mastitis, as complement activation at the blood–milk barrier contributes to bactericidal activity and neutrophil recruitment during infection (Rainard and Riollet, 2006). The downregulation of complement pathway genes in IL10RA-KO cells infected with SCV suggests that IL10RA may normally facilitate complement-mediated defense mechanisms in the mammary epithelium, and that its loss would impair this arm of innate immunity during chronic SCV infection.

The downregulation of the Hippo signaling pathway among WT-SCV downregulated DEGs (**Figure 3B**) and its upstream regulator-associated pathways in IL10RA-KO conditions collectively suggests that SCV infection, with or without IL10RA, suppresses epithelial tissue homeostasis and organ-size regulation pathways. The Hippo pathway, acting through its effectors YAP and TAZ, is central to epithelial regeneration, cell proliferation, and barrier maintenance (Yu and Guan, 2013). Suppression of Hippo signaling during persistent intracellular SCV infection may therefore compromise mammary epithelial regenerative capacity, contributing to the chronic tissue damage and reduced milk production characteristic of subclinical *S. aureus* mastitis (Halasa et al., 2007).

### Comparison with published transcriptomic studies and broader implications

The present findings situate within a growing body of transcriptomic evidence from *S. aureus*-infected bovine mammary epithelial cells. Chen et al. (2021) reported that RNA-seq analysis of exosomes from *S. aureus*-infected MAC-T cells identified DEGs enriched in MAPK, NF-κB, p53, TNF, and Hippo signaling pathways, a pattern strikingly consistent with the enrichment observed in our IL10RA-KO infection conditions. Similarly, Lin et al. (2021) demonstrated that *S. aureus* infection of MAC-T cells triggered downregulation of lncRNA target genes enriched in cell adhesion molecules, PI3K-Akt signaling, and bacterial invasion pathways, which are also disrupted in our IL10RA-KO conditions. Wang et al. (2021) conducted an *in vivo* integrative analysis of miRNA and mRNA profiles in mammary glands of Holstein cows infected with *S. aureus*, identifying 1,625 DEGs enriched in PI3K-Akt, TNF, HIF-1, IL-17, and Rap1 signaling pathways. The IL-17 and TNF signaling enrichments observed in our IL10RA-KO infection conditions are consistent with these *in vivo* findings, suggesting that the transcriptional programs derepressed by IL10RA ablation *in vitro* partially recapitulate those activated in the infected bovine mammary gland *in vivo*.

Zhao et al. (2025a) recently reported that *S. aureus* infection of MAC-T cells induced 354 DEGs including pathogen-specific upregulation of ESM1 and IL18RAP, the latter indicating NLRP3 inflammasome activation. While our study used an isogenic SCV, the broadly similar gene counts between WT-SCV (461 DEGs) and Zhao et al.’s prototypic *S. aureus* strain suggest comparability in the magnitude of WT epithelial transcriptional responses. The substantially greater DEG amplification in IL10RA-KO-PS (619 DEGs) and IL10RA-KO-SCV (1,297 DEGs) conditions relative to WT therefore underscores the quantitatively dominant regulatory role of IL10RA in constraining epithelial immune gene expression during staphylococcal infection.

The comparison between our IL10RA-KO findings and the parallel TLR4-KO study (Shandilya et al., 2021) reveals both shared and divergent regulatory functions of these two receptor systems. Both IL10RA and TLR4 disruption amplified transcriptional dysregulation, though to different magnitudes (192-fold increase for TLR4-KO-PS vs. 77.4-fold for IL10RA-KO-PS). Both systems regulated shared structural genes, including cytoskeleton organization, cornified envelope formation, and extracellular matrix pathways. However, TLR4-KO uniquely produced a ~57% DEG overlap between PS and SCV conditions, indicating a dominant role in strain-specific transcriptional discrimination, while IL10RA-KO produced a more modest ~32–36% overlap, reflecting a quantitatively different regulatory architecture. The novel upregulation of *WFDC2* in both KO systems is a particularly compelling convergence point, suggesting that WFDC2 induction represents a universal compensatory innate immune response activated by disruption of either pro- or anti-inflammatory receptor signaling at the bovine mammary epithelial surface.

### IL10RA polymorphisms and translational relevance to bovine mastitis susceptibility

The mechanistic findings reported here have direct translational relevance to bovine mastitis genetics. Several studies have identified single-nucleotide polymorphisms (SNPs) in the IL10RA gene associated with differential mastitis susceptibility in dairy cattle. Ibeagha-Awemu et al. (2008) identified IL10RA variants associated with somatic cell count and mastitis occurrence in Canadian Holstein cows, while subsequent genome-wide association studies have repeatedly identified the IL-10 signaling axis as a candidate pathway in mastitis resistance QTL (Bloemhof et al., 2009). If natural IL10RA variants alter the receptor’s signaling efficiency, analogously to the complete ablation modelled here, the transcriptional consequences could encompass dysregulation of hundreds of genes governing epithelial inflammatory balance, barrier integrity, and bacterial clearance. The identification of AMN, PGAP4, LY6E, ZDHHC14, CD79B, WFDC2, MYT1, and NOSTRIN in the present study should be interpreted as hypothesis-generating rather than as evidence of validated biomarkers of mastitis susceptibility. Because these findings were generated using an *in vitro* IL10RA-KO MAC-T cell model, further validation in primary bovine mammary epithelial cells, mammary secretions, milk somatic cells, or biopsy samples from cows with contrasting mastitis phenotypes will be required before these genes can be considered diagnostic biomarkers or therapeutic targets. Although RNA-seq provides robust genome-wide evidence for IL10RA-dependent transcriptional reprogramming, future studies will validate representative genes from the major functional categories highlighted in this study, including inflammatory signaling, phagosomal function, lipid metabolism, and highly regulated candidate genes such as *CD79B, AMN, WFDC2*, and *LY6E*, using RT-qPCR across independent biological replicates. In addition, protein-level validation of unexpected targets, particularly PD-1 and CD79B, using immunoblotting or immunofluorescence staining will be necessary to determine whether the observed transcript-level changes translate into corresponding protein expression.

## Conclusion

Taken together, the present study provides the first comprehensive transcriptomic characterization of IL10RA-dependent host responses in bovine mammary epithelial cells challenged with *S. aureus* PS and its isogenic SCV. Our findings demonstrate that WT MAC-T cells mount fundamentally distinct, strain-specific transcriptional responses to PS and SCV infection, with the SCV eliciting a markedly broader response characterized by lipid metabolic reprogramming and suppression of epithelial barrier integrity genes consistent with its immune-evasive, persistence-promoting phenotype. IL10RA emerges as a critical regulator of epithelial transcriptional homeostasis and inflammatory balance during staphylococcal challenge: its disruption dramatically amplifies pro-inflammatory cytokine signaling (TNF, IL-17, cytokine–cytokine receptor interaction), derepresses apoptotic programs during SCV infection, and suppresses phagosomal maturation, lipid metabolic, and structural homeostasis gene programs. A moderate core set of IL10RA-regulated genes (~32–36%) is shared across infection strains, with the SCV condition driving a substantially greater proportion of strain-specific transcriptional change. These findings enrich our molecular understanding of chronic bovine mastitis pathogenesis, implicate IL10RA as a broad transcriptional homeostasis factor in mammary epithelial cells beyond its classical anti-inflammatory function, and identify *CD79B*, *WFDC2*, *AMN, PGAP4, NOSTRIN*, and *LY6E* as candidate genes warranting further validation in primary bovine mammary cells and clinical mastitis samples.

## Data Availability

The data presented in the study are deposited in the Gene Expression Omnibus database under accession number GSE334081.
